# Abstract of Proceedings of Buffalo Medical Association

**Published:** 1869-08

**Authors:** T. M. Johnson

**Affiliations:** Secy.


BUFFALO
Medical and Surgical Journal.

VOL. IX. AUGUST, 1869. No. 1.

### Original Communications.

ART. 1.—*Abstract of Proceedings of Buffalo Medical Association.*

A Special Meeting of the Association was held on Thursday evening, July 22d, at which the Resolutions passed by a meeting of Erie County Medical Society, of the same date, in relation to the death of DR. J. R. LOTHROP, were unanimously adopted.

*Stated Meeting, Tuesday Evening Aug. 3d,* 1869.

The chair was occupied by Dr. J. F. Miner, President.

Members Present—Drs. Miner, Samo, Gay, Abbott, Taylor, Phelps, Wetmore, Cronyn, Diehl, Kamerling and Johnson.

The subject of admission to membership being in order, the application for membership of Dr. Wm. J. Talbott was introduced. The Secretary read a letter from the applicant, which, by vote, was accepted and placed on file. Nearly every member present spoke in disfavor of admitting Dr. Talbott to membership, because they believe that he has, until quite recently, been practising in an irregular and quackish manner, and is not entitled to their confidence.

Dr. Samo moved that the application of Dr. Talbott be laid on the table. The motion was carried unanimously.

Dr. W. O. Taylor reported the following case for Dr. James P White:—

Mrs. B———, aged 26, has had one child, which is living; five months ago had an abortion which was followed by hemorrhage, low fever, peritonitis and an abscess in either labia majora. July 17th, the patient, by advice of her attending physician, Dr. Benedict of Dunkirk, applied to Dr. White, of Buffalo, for relief from some undetermined uterine trouble. On examination Dr. White found complete atresia of the vagina. Introducing the finger into the rectum a large tumor could be distinguished, moderately soft and apparently containing fluid, probably retained menstrual fluid.

The doctor succeeded in introducing the finger a short distance into the vagina, breaking down firm adhesions as he proceeded; some hemorrhage followed, and further interference was deferred, and the vagina plugged with cloths saturated with glycerine.

July 18th—Patient comfortable, Dr. White with Drs. Potter and Taylor as assistants, placed the patient on the side of the bed and administered chloroform. The doctor again endeavored to introduce the finger within the vagina. The adhesions connecting the vaginal walls yielded to continued and strong pressure by the finger, and the lower portion of the tumor, which proved to be the distended uterus, was reached. The uterus was then firmly grasped and pushed downwards by Dr. Potter while Dr. White pushed the right fore-finger through the occluded os uteri externum. On withdrawal of the finger, from 26 to 30 ounces of thick, dark red, pitchy fluid escaped. The uterus was then washed out with warm water, the vagina plugged with cloths soaked in a solution of glycerine and liq. ferri persulphatis. A compress was applied over the uterus, secured by a bandage around the pelvis, and half gr. doses of sulphate of morphia prescribed.

July 19th—The patient was doing well. Since this date she has gradually improved, and on Saturday, July 31st, she returned to her home. She was directed to wear a glass dilator to prevent contraction of the vagina.

August 14th, the patient returned to Buffalo. Her health had improved very much. The genital canal was entirely free from obstruction, and she was menstruating freely without pain. The dilator she had been using was exchanged for one larger.

On examination per vaginam, after the evacuation of the fluid, the cavity of the uterus appeared to be almost continuous with that of the vagina; the thin flabby cervix was pressed against the vaginal circumference, and the lips of the widely distended os tincæ imparted to the touch the sensation of a soft membrane, standing out two or three lines from the vaginal walls, with an irregular torn edge,yielding in any direction to the slightest pressure. Dr. White considered the uterus hypertrophied, although the uterine parieties were very much attenuated, owing to the distended condition of the cavity of this organ. In other words, the weight was greater than that of an ordinary uterus, although the walls were much thinner than usual. The temperature of the cavity was probably not above the normal temperature of cavities in the body excluded from the atmosphere.

The above is a rare and typical case of true hæmatometra of the uterus, produced by atresia of the entire vaginal canal, and occlusion of the os tincæ, these lesions most probably originating in inflammation at or after the time of the above mentioned abortion.

Klob, in his Pathological Anatomy of the Female Sexual Organs gives this definition. "By hæmatometra, strictly speaking, we understand an accumulation of blood in the non-gravid uterus." Elsewhere he goes on to say, "according to the amount of the accumulations the uterus is distended in various degrees, and after this has continued for a length of time, its walls are almost always hypertrophied. On section, the substance of its walls appears pale, often milky white, congested and peculiarly soft, but still resistant. After the uterus has been considerably distended, a thinning of its walls becomes apparent, yet the amount of its substance is much greater than in its normal state. The accumulated blood is mostly dark, deficient in film, discolored in various degree to a blackish brown, thickened rather than coagulated, tar-like, and sometimes mixed with crystals of cholesterine." This is a most graphic description of the appearance of the fluid in Dr. White's case, and tends also to show how careful and exact an observer is Prof. Klob. The chief danger apprehended in this case was inflammation, and especially purulent infiltration of the uterine tissue, which would probably have been followed by thrombosis, septæmia, etc.

Graily Hewitt in treating of hæmatometra states that "accumulation of fluids in the uterus is associated with closure of the outlet, narrowing and stricture of the cervix, agglutination of the os uteri, flexion of the uterus, presence of a tumor in the cervix or lower part of the uterus," and adds, "The importance of retroflexion as leading to slight accumulations of fluid in the uterus, is not sufficiently recognized.

Kruner mentions that hypertrophy of the vaginal portion of the cervix may also lead to a contraction of the cervical canal, and thus hinder the escape of menstrual blood.

Klob states that "hæmatometra is generally caused by the collection of the menstrual blood, in consequence of atresia of the genital canal. These atresiæ occur most frequently in the vagina from congenital or acquired causes, or from imperforation of the hymen."

The case reported is certainly of rare occurrence, the entire vaginal canal with the external os uteri being obliterated. In still rarer instances the obliteration extends as far as the internal os.

We see then that any obstruction to the free egress of menstrual blood may give rise to hæmatometra in greater or less degree. The treatment is obvious—the great desideratum being to evacuate the the contents of the uterus. A few words with regard to atresia of the vagina will not be out of place here. Dr. Gaillard Thomas of New York mentions the following, as causes of this pathological condition: "Arrest of developement, prolonged and difficult labor, chemical agents locally applied, mechanical agencies, sloughing, the result of impaired vitality, syphilitic or other extensive ulcerations." Inflammation occurring after child-birth or abortion seems to be a frequent cause.

In the proceedings of Buffalo Medical Association, Nov. 5th, 1861, Dr. White reported a case of adhesion of the lips of the uterine outlet, caused by mechanical irritation, occasioned by an attempt to procure abortion, and later at a meeting of the same society, Dec. 5th, 1865, a case of partial adhesion of the vaginal walls, and complete obliteration of the cavity of the cervix uteri. In this case the woman was delivered by instruments, inflammation supervened and was the cause of the atresia. Occlusion of the cavity of the cervix, according to Klob, is of exceedingly rare occurrence.

After operations for the relief of atresia, the surgeon is often troubled by the recurrence of the abnormal condition, or by the cicatricial contraction, causing stricture of the vagina. Thomas recommends in order to prevent this, that a glass plug be worn for some time. This plug, commonly known as Sims' vaginal dilator, Dr. Sims has been in the habit of using after his operation for vaginismus, and he holds that it not only prevents contraction of the cicatrix but also facilitates the healing process.

Emmet believes when the incisions are made by scissors or the tissue torn by the fingers, subsequent contraction and atresia are much less likely to occur. Graily Hewitt uses the finger alone if practicable. And in Dr. White's case the adhesions were broken down by the finger, the result of the operation which was most satisfactory, proves this to be an excellent method of procedure.

Dr. Miner said he would call attention to one danger not mentioned by Dr. Taylor from such accumulation in the uterus; not danger from the operation so much as from the disease.— So far as his observation warranted a conclusion, absorption of pus, acute inflammation following the operation and contraction of the orifice, were remote causes of failure. The uterus, after distension from such unnatural cause, or its lining membrane, from such direct exposure to the air, would sometimes take on the properties, in some degree at least, of pyogenic or pus secrecting membrane, and would furnish a continuous and exhausting discharge, which would finally prove fatal.

The interesting report furnished by Dr. Taylor reminded him of a case in some respects similar, but in some features rare and remarkable. Dr. Tobie, of this city, a few months since, had under his care a young German girl, about sixteen years of age, at this time quite healthy in appearance and well developed. The patient had complained, for several months, of most severe pain, lasting five or six days and appearing regularly every four weeks. The abdomen was enlarged to about the size of pregnancy at the sixth month. Upon examination, Dr. Tobie discovered that there was no vagina; that the space between the urethra and rectum was occupied with dense fibrous unyielding tissue; that the uterus was distended with fluid; and believe that the pain was due to the monthly secretion and increase of fluid. He now proposed an operation for relief; and I was associated with him in an operation for establishing an artificial vaginal outlet for the menstrual fluid. The patient being fully anæsthetised, an incision was made in the site of the vagina, dividing the fibrous and muscular tissues with the scalpel to the depth of one and a half or two inches, the finger assisting to dilate and extend the opening. A large trocar and cannula was now passed into the womb, and the diagnosis fully confirmed. The grumous, dark, tenacious, gluey fluid, was too thick to pass through the cannula, and the instrument was withdrawn. The finger now dilated the opening, extending it in all directions, so that the fluid gradually escaped. About two quarts, according to recollection, was obtained, and the distended abdomen relieved. The uterus contracted down to more natural proportions, but how perfectly was unable to state. The patient was relieved of former pains, but another train of symptoms appeared. There has been an exhausting muco-purulent discharge, which appears likely to continue until it produces a fatal termination. Symptoms of general disease are also present.

He had related the main features of the case as illustrating a source of danger after such operations; but, aside from this, the case had features of great interest. This was a congenital malformation of great rarity—probably only a few similar cases are on record. It is not very uncommon for the os uteri to become occluded; inflammation of the os and neck may, and quite frequently does, result in closure of the canal. The vagina also becomes occluded in something the same way. Inflammation of its walls results in partial or even complete closure. Complete absence of vagina, neck and os uteri, must certainly be rare.

The great difficulty of establishing these outlets is apparent to all surgeons. It requires long and patient effort, and even then is often attended by failure. The surgeon is unable to transplant any thing to serve the purpose of the mucous membrane, by which such surfaces are lined and prevented from uniting in the normal condition, and thus the almost certain contraction, agglutination and final closure. As to the comparative merits of the various plans which have, from time to time, been proposed, he could add nothing of importance to the remarks of Dr. Taylor. It was probable that no plan yet proposed was quite satisfactory, and that contraction, and sometimes re-union, would be the result, however well directed, might be the plan of operation, or faithful the after treatment.

Dr. Phelps said that he assisted Dr. Hill to operate upon a patient in whose vagina existed a fleshy partition extending transversely across the cavity like a diaphragm: A bistoury was passed through it, when a large amount of grumous matter passed away. This lady had no children by her first husband, with whom she had lived several years. She was, at the time of the operation, living with her second husband, and became pregnant a few months after the operation. The abnormity had existed before her first marriage.

Dr. Cronyn remarked that the class of cases reported by Drs. Taylor and Phelps were, comparatively, quite common; but that the case reported by Dr. Miner was of very unusual occurrence and of much interest. He had recently seen a very unusual case, which was that of a large masculine woman with no perceptible uterus. He had made a careful examination, and found a vagina four and a half inches in length, but no indication of a uterus. All other appearances were those of an unusually large sized, coarse woman. Upon investigation, he was satisfied that the abnormity was congenital. There never had been any indications of menstruation.

Adjourned.

T. M. JOHNSON, *Secy.*

